# Supplementary material for: Identification of photoperiod-induced specific miRNAs in the adrenal glands of Sunite sheep (Ovis aries)
Source: Front Vet Sci. 2022 Jul 22;9:888207. doi: 10.3389/fvets.2022.888207 (PMC9354845; doi:10.3389/fvets.2022.888207)
Supplement: Supplementary file 1 [file Data_Sheet_1.ZIP › Supplementary file/miRDeep2 file.zip!/miRDeep2 file/Know_Structure/LP42A1/oar-mir-199a.pdf]

miRBase precursor : oar-mir-199a
Total read count : 140380
oar-miR-199a-3p read count : 90061
remaining reads : 50319

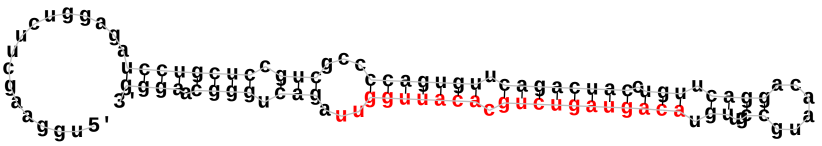

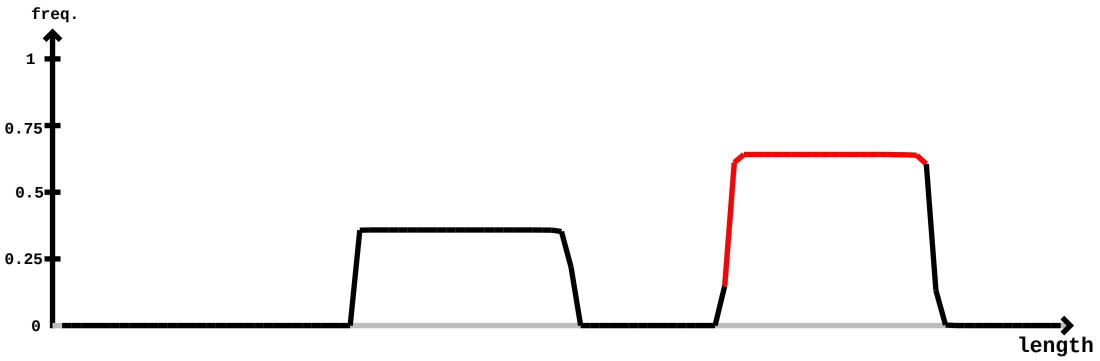

oar-miR-199a-3p

5'- uggaagcuucuggagauccugcuccgucgccccaguguucagacuaccuguucaggacaaugccguuguacaguagucugcacauugguuagacugggcaaggg -3' exp

.................(((((((.(((...(((((((.((((((((.(((.((((......))..)).))))))))))).)))))))...))).)))).))).	reads	mm	sample
.............................ccccaguguucagacuaccuguu..	5	0	seq
.............................ccccaguguucagacuaccuguuc...	1	0	seq
..............................cccaguguucagacuac...	1	0	seq
..............................cccaguguucagacuacc..	2	0	seq
..............................cccaguguucagacuaccu...	38	0	seq
..............................cccaguguucagacuaccug..	45	0	seq
..............................cccaguguucagacuaccugu...	729	0	seq
..............................cccaguguucagacuaccuguu..	18644	0	seq
..............................cccaguguucagacuaccuguuc...	30720	0	seq
...............................ccaguguucagacuaccugu...	4	0	seq
...............................ccaguguucagacuaccuguu..	43	0	seq
...............................ccaguguucagacuaccuguuc...	82	0	seq
................................caguguucagacuaccuguu..	1	0	seq
................................caguguucagacuaccuguuc...	2	0	seq
..................................guguucagacuaccugCu..	1	1	seq
....aggacaaugccguuguacaguagucugca......................	1	0	seq
....uacaguagucugcacau...................	3	0	seq
....uacaguagucugcacauu..................	43	0	seq
....uacaguagucugcacauug.................	12	0	seq
....uacaguagucugcacauugg................	132	0	seq
....uacaguagucugcacauuggu...............	2302	0	seq
....uacaguagucugcacauugguu..............	16748	0	seq
....uacaguagucugcacauugguua.............	1457	0	seq
....uacaguagucugcacauugguuag............	17	0	seq
....acaguagucugcacauu..................	79	0	seq
....acaAuagucugcacauu..................	1	1	seq
....acaCuagucugcacauu..................	3	1	seq
....acaguagucugcacauug.................	21	0	seq
....acaguagucugcacauugg................	138	0	seq
....acaguagucugcacauuggu...............	2089	0	seq
....acaguagucugcacauugguu..............	46906	0	seq
....acaguagucugcacauugguua.............	15622	0	seq
....acaguagucugcacauugguuag............	235	0	seq
....acaguagucugcacauugguuagG...........	3	1	seq
....acaguagucugcacauugguuaga...........	20	0	seq

oar-miR-199a-3p

uggaagcuucuggagauccugcuccgucgccccaguguucagacuaccuguucaggacaaugccguuguacaguagucugcacauugguuagacugggcaaggg			
....caguagucugcacauug.................	4	0	seq
....caguagucugcacauugg................	8	0	seq
....caguagucugcacauuggu...............	188	0	seq
....caguagucugcacauugguu..............	2863	0	seq
....caguagucugcacauugguua.............	1111	0	seq
....caguagucugcacauugguuag............	29	0	seq
....caguagucugcacauugguuaga...........	1	0	seq
....aguagucugcacauugg................	1	0	seq
....aguagucugcacauugguu..............	17	0	seq
....aguagucugcacauugguua.............	8	0	seq
